# What makes the pipeline leak? Women’s gender-based rejection sensitivity and men’s hostile sexism as predictors of expectations of success for their own and the respective other gender group

**DOI:** 10.3389/fpsyg.2022.800120

**Published:** 2022-10-04

**Authors:** Karen Ollrogge, Malte Roswag, Bettina Hannover

**Affiliations:** ^1^Department of Education and Psychology, Freie Universität Berlin, Berlin, Germany; ^2^Department of Psychology, University of Hildesheim, Hildesheim, Lower Saxony, Germany

**Keywords:** leaky pipeline, gender, academia, expectations of success, rejection sensitivity, hostile sexism

## Abstract

In academia, the proportion of women decreases with each career level. In this research, we examined how this so-called leaky pipeline relates to gender-based relative expectations of success. The participants were students from social sciences where women are the majority among students, such that it is more readily – but erroneously – inferred that gender discrimination is not an issue. We assumed that gender-based relative expectations of success should be predicted by two variables. Women students should experience higher gender-based rejection sensitivity than men students, with gender-based rejection sensitivity mitigating relative success expectations in women, but not in men. Men students should exhibit higher hostile-sexist attitudes toward women than women students, with hostile sexism reducing men students’ but not women students’ relative success expectations. We tested our hypotheses in an (under-)graduate sample of women and men students enrolled in educational or psychological majors (*N* = 372). Results show that a quarter of the women students expected men to be more successful than women and that proportionately more women than men students indicated that women have worse chances of success than men in the job they aspire to. Women were more concerned about being treated differently because of their gender than men, and men held more sexist attitudes toward women than women, with gender-based rejection sensitivity contributing to women students’ and sexism to men students’ expectation that their own gender group will less likely succeed in their aimed for future job. Implications how the leaky pipeline can be patched are discussed.

## Introduction

In academia, the proportion of women decreases with each career level, a phenomenon for which Clark Blickenstaff (2005) coined the term *leaky pipeline*. An example are higher education institutions where from the undergraduate to the professorial staff level the percentage of women is declining steadily (e.g., for the United Kingdom: Cooper, 2019; for Germany where our study was conducted: German Federal Office of Statistics, 2019). Contributing to the current Research Topic, we investigated the social sciences. The study of psychological causes of the leaky pipeline is particularly interesting for this domain: as women are the majority among students in the social sciences and are also relatively well represented at lower hierarchical levels within the academic staff, the persistence of gender discrimination – as shown in the leaky pipeline – is less obvious than in domains where women are underrepresented at all levels, such as in the fields of natural sciences and technology. This can prove to be an additional disadvantage for women seeking careers within fields where women are well represented on average. For instance, investigating a discipline in which women professionals had a share of 50% + for more a decade now, veterinary medicine, Begeny et al. (2020) found that women still experienced greater discrimination and less recognition from colleagues than men. In an experimental study, Begeny et al. (2020) found that managers evaluated the performance review of a vet called Mark as more competent and suggested a higher salary – equating to an 8% gender pay-gap – than when they assessed the same performance review of an employee called Elizabeth. But not only negative stereotypes toward women, such as that they are less competent, also (apparently) positive stereotypes may play a role even in academic domains in which women are well represented on average. While negative stereotypes are largely considered inappropriate today, people may emphasize a group’s positive traits – without experiencing themselves as prejudiced or being perceived as prejudiced by others (Czopp et al., 2015). Even when expressed with benevolent intent, positive stereotypes (e.g., women as warm and caring; Eagly et al., 1991) can have the same adverse effects on targeted individuals as negative stereotypes, namely self-stereotyping and feeling of depersonalization due to being acknowledged through one’s group membership rather than one’s personal achievements (for a review see Czopp et al., 2015). Taken together, these findings suggest that gender stereotypes persist even in disciplines where the overall percentage of women is high and that since gender stereotypes’ existence is less obvious, they have significant psychological consequences. In line with this view, van Veelen and Derks (2022) found that in the social sciences – but not in natural sciences, technology, and economics – women assistant and associate professors perceived a thicker glass ceiling than their men colleagues and considered it less likely to become full professors the thicker they perceived the glass ceiling to be (with no such moderated mediation appearing for men).

van Veelen and Derks (2022) had their research participants estimate the likelihood that they will become a full professor during their career. Such expectations of success are an empirically well-established predictor of achievement-related choices and persistence in academia. As Muenks et al. (2018) point out in their literature review, the concept of expectancy-related beliefs is found in numerous motivational theories, such as expectancy-value theory, social cognitive theory, and theories on self-concept and self-worth. A common feature of the various approaches is the assumption that individual differences in the choice of task difficulty, in engagement and persistence in the pursuit of a goal can be explained by how strongly the person is convinced that they can succeed, i.e., by the person’s expectations of success. In our study we investigated university students’ gender-based expectations of success regarding the future profession they aimed to work in. More specifically, we asked them whether they thought that women have worse, the same, or better chances of success than men in their aimed for future job, assuming that subjective chances of success matter for students motivationally.

### Women students’ gender-based relative expectations of success

With gender discrimination remaining an issue even in disciplines where women are well represented on average (e.g., Begeny et al., 2020; van Veelen and Derks, 2022), we expected that women students in the social sciences often have pessimistic expectations regarding their gender group’s future success in the jobs they personally aspire to. If gender and gender stereotypes do not play a role, individuals’ real chances of success should depend on their performance and other idiosyncratic personal characteristics. Accordingly, men and women should have equal chances on average. We asked our participants whether men and women have (a) the same, (b) relatively better, or (c) relatively worse chances in the profession they themselves aim to work in. While respondents who consider gender to be a non-significant predictor of success should choose response option (a), we assumed for respondents who consider women to be disadvantaged that they choose response option (b) and for respondents who consider men to be disadvantaged that they choose response option (c). For women students, we predicted a pessimistic expectation. That is, the proportion of women who believes that men are more likely to succeed than women should be greater than the proportion who believes that women are more likely to succeed than men, and proportionately more women than men students should expect that women have worse chances of success than men in the job they aspire to.

We further assumed that among women students, the pessimistic expectation that women have lower chances of success in their future job than men do is predicted by gender-based rejection sensitivity. Gender-based rejection sensitivity is a cognitive–affective process triggered by the personal experience or the witnessing of other ingroup members to be discriminated against or socially excluded due to gender (London et al., 2012). Such experiences make the person anxiously expect to be rejected even by unfamiliar other persons or in newly encountered situations, to monitor new contexts for possible rejections, heighten their readiness to perceive rejection, and intensify their emotional reactions to rejection (Mendoza-Denton and Goldman-Flythe, 2009; London et al., 2012; Ahlqvist et al., 2013). We expected that women noticing gender disparities on higher career levels are inclined to experience gender-based rejection sensitivity and accordingly expect men to have higher success in their future job than women.

Regarding gender-based rejection sensitivity, we expected that due to the experience of women’s limited advancement to higher career levels, women students are more sensitive to gender-based discrimination than their men fellow students. We further assumed that this difference would be particularly pronounced in encounters with men staff members or peers, as gender is more salient in mixed gender groups than when only one gender is present (e.g., McGuire and Padawer-Singer, 1976; Kessels and Hannover, 2008). Also, a woman getting treated in a discriminatory manner by a man because of her gender represents a *prototypical situation* of discrimination and therefore concerns of gender disadvantage should be inherent to any interaction of a woman with a man (Carlsson and Sinclair, 2018).

### Men students’ gender-based relative expectations of success

Regarding the dependency of men students’ rejection sensitivity on the interaction partner’s gender, our expectations deviated from what we had assumed for women students. As gender is more pronounced in mixed gender than in same-gender encounters (*cf.* McGuire and Padawer-Singer, 1976; Kessels and Hannover, 2008), it could be argued that both women and men students are more anxious when interacting with someone of the other gender group. However, men students witness women’s limited advancement to higher career levels in their own study environment. This could mean that men students experience women as less powerful than men and accordingly do not feel more threatened to be discriminated against based on their own gender in an interaction with a woman than in an interaction with a man. We therefore expected men students’ gender-based rejection sensitivity to be the same, irrespective of the interaction partner being a woman or a man.

For men, the perception of the leaky pipeline should imply that their own gender group has good career prospects – even though they are in the minority among students. This leads to the prediction that the proportion of men students who think that men have better chances of success in their future job than women is larger than the proportion of men who think women’s relative success is greater than men’s.

At the same time, however, research found men and women to be particularly sensitive toward discriminatory treatment of members of their own gender group (Elkins et al., 2002). We therefore considered it also possible that men too – mirroring women’s gender-related relative expectations of success – would be more likely to expect their own gender group’s career opportunities to be lower than those of the other gender group.

In either case, however, men’s gender-based success expectations should be unrelated to gender-based rejection sensitivity. The prototypical situation of gender-based discrimination is one in which a woman is disadvantaged by a man or by men, with the prototypicality of a situation influencing how likely people experience or perceive the interaction as discriminatory (Carlsson and Sinclair, 2018). Hence, we predicted that in men, gender-based rejection sensitivity would be unrelated to their gender-based relative expectations of success.

We examined hostile sexism toward women as a predictor that should inversely predict in men, but not in women, how they view the relative career opportunities of men and women. Hostile sexism is an overtly negative attitude characterized by the belief that women are inferior, incompetent, and trying to control men or take advantage of them (Glick and Fiske, 1996). Many studies have shown that men endorse hostile sexism toward women to a stronger extent than women do (e.g., Glick et al., 2000; Cowie et al., 2019). In our study, we expected hostile sexism to predict men students’ expectation that women would (unjustifiably) be given better opportunities in their future job than men, while hostile sexism should be unrelated to gender-based relative expectations of success in women students.

### The present study

In an online survey with students of the social sciences, we measured gender-based rejection sensitivity, hostile sexism toward women, and gender-based relative expectations of success for own and the respective other gender group and tested the following hypotheses:

1. The proportion of women students who believe that men are more likely to succeed than women is greater than the proportion who believes that women are more likely to succeed than men.
2. For men students, no directed hypothesis was specified regarding their gender-based relative expectations of success. It is possible that the proportion of men students who think that men have better chances of success in their future job than women is larger than the proportion of men who think women’s relative success is greater than men’s, or vice versa, or that they are equal.
3. The proportion of women students who expect women to be less successful than men in the aspired for future job is larger than the proportion of men students.
4. Gender-based rejection sensitivity is stronger in women than in men students.
5. While women students are particularly anxious to be discriminated based on their gender in encounters with a man, for men students the interaction partner’s gender does not matter.
6. Men students endorse hostile-sexist attitudes toward women more strongly than women students.
7. Rejection sensitivity predicts gender-based relative expectations of success in women but not in men. This should also become evident in gender moderating the relationship between rejection sensitivity and expected success.
8. Hostile sexism predicts gender-based relative expectations of success in men but not in women. This should also become evident in gender moderating the relationship between sexism and expected success.

## Materials and methods

### Participants

The online study took place at a large German university and was part of a more comprehensive survey examining experiences of sexual harassment and violence. All measures reported in this paper were collected prior to the sexual harassment and violence survey. Three hundred and eighty-four students of the social sciences (educational science, teacher education, psychology) participated. The university’s ethics committee approved the study under the constraint that we were not allowed to collect any personal data besides gender, in order to ensure anonymity even for those who identify as non-binary. Of the participants, 311 identified as women, 63 as men, 6 as non-binary, and four individuals did not specify their gender. This corresponds to the ratios of the genders as they are in the social sciences at the university studied here, *χ*^2^(1) = 1.96, *p* = 0.16. While 221 students were enrolled in bachelor studies, 161 students were pursuing their master’s degree. One person was pursuing another degree, and one person did not indicate the degree. According to the enrollment office, at this university the age of students in the social sciences is on average 26.31 (*SD* = 6.67). We were also able to receive information from the enrollment office that 11.1% of the students in the subjects we examined were not born in Germany. Of these, 27.9% have German citizenship. The most frequently represented countries of origin of students with a migration background in our sample were Turkey, Russia, China, and the US.

### Measures

Participants completed measures of gender-based rejection sensitivity, hostile sexism toward women, and gender-based relative expectations of success. To measure gender-based rejection sensitivity, we adapted the scale of London et al. (2012) by choosing all situations that fit well to the scenario of studying at a university. In total, we extracted six situations (out of 11) where gender rejection may be experienced. We worded all items in such a way that respondents should relate them to their own field of study (e.g., “Imagine that you have to give an oral presentation in a very important course. After everyone gives their presentations, the professor announces that he/she will post the grades outside of the classroom.”). We translated the situations into German, and an independent native English speaker translated them back into English. In the grammatical gender language German, nouns and adjectives are gendered (e.g., for a woman professor: Professorin, for a man professor: Professor). Therefore, for each of the six items we developed one version in which the acting person was a woman (e.g., Professorin P.) and one in which she was a man (e.g., Professor P.). We then created two different blocks. Block A: in situations 1, 3, and 5 the acting person was a man and in situations 2, 4, and 6 a woman; Block B: in situations 1, 3, and 5 the acting person was a woman and in situations 2, 4, and 6 a man. Each participant was randomly assigned to one block.

Following the procedure of London et al. (2012), for each situation, participants rated their level of concern about being rejected or treated unfairly because of their gender on two 6-point Likert scales: (1) “How concerned would you be that you would be treated differently or have a negative experience because of your gender?” and (2) “To what extent would you expect to be treated fairly?” (response scales: 1 = *not at all*, 6 = *very strongly*). Again, following the procedure by London et al. (2012), responses to items 2 were reverse coded, and for each situation, the item 1-score was then multiplied by the item 2-score, such that higher product-scores reflect stronger rejection sensitivity. Product-scores were averaged across the six situations, with the resulting rejection sensitivity score ranging between 1 and 36. The Cronbach’s alphas across the six product-scores of the gender-based rejection scale was good with a total value (averaged across Block A and B) of 0.85 and a value of 0.81 for the man acting person and 0.80 for the woman acting person. Hostile sexism toward women was measured with a subscale of the ambivalent sexism inventory (original: Glick and Fiske, 1996; German translation: Eckes and Six-Materna, 1999). The hostile sexism subscale measures overtly hostile attitudes toward women. Participants responded to 11 statements, such as “Women are too easily offended,” on six-point Likert scales (1 = *strongly disagree*, 6 = *strongly agree*). The reliability for hostile sexism was good with a Cronbach’s alpha of 0.91. Regarding gender-based relative expectations of success, students were asked to indicate whether men or women have better chances to be successful in their intended future job. Responses were given on a three-point scale (“Women have worse chances of success than men in the job I aspire to”; “Women have the same chances of success as men in the job I aspire to”; “Women have better chances of success than men in the job I aspire to”).

## Results

**Figure 1:**
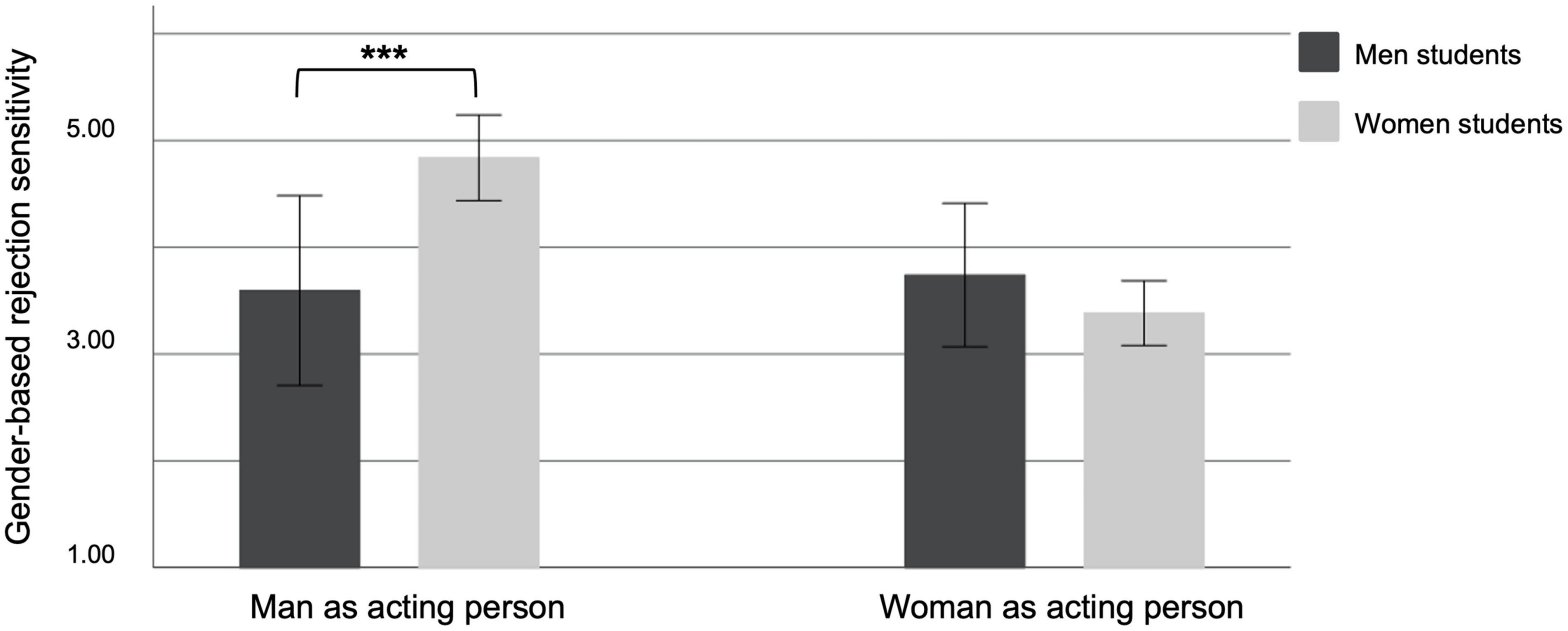
Gender-based rejection sensitivity depending on the acting person’s gender and of participants’ gender. Depicted are mean total scores with 95% confidence intervals of the gender-based rejection sensitivity scale. Asterisks highlight significant between-group differences. ^***^p < 0.001.

Only participants who identified as women or as men were included in all further analyses. Data was analyzed using SPSS 25. Since the variance homogeneity assumption for the *t*-test was violated, we performed a Welch-test to test Hypothesis 6 regarding the gender difference in hostile sexism. A sensitivity analysis indicated that this test would be sensitive to effects of Cohen’s *d* = 0.39, given a sample size of 63 men and 309 women students (*α* = 0.05, two-tailed). This means our study would not be able to reliably detect effects smaller than Cohen’s *d* = 0.39. As expected, men students reported more hostile sexism (*M* = 2.35, *SD* = 1.1) than women students (*M* = 1.86, *SD* = 0.74), *t*(73.66) = 3.35, *p* = 0.001, *d* = 0.6. To test our hypotheses regarding gender differences in gender-based rejection sensitivity, we conducted a repeated measurement ANOVA with gender of the student as between-participant-factor (Hypothesis 4) and gender of the acting person (man vs. woman) as within-participant-factor (Hypothesis 5). A sensitivity analysis indicated that effects of *η*_p_^2^ = 0.02 could be detected with a sample size of 373 students and a power of 80% (*α* = 0.05, two-tailed). As expected, a significant main effect for student gender was found, *F*(1, 371) = 11.86, *p* = 0.001, *η*_p_^2^ = 0.031, with women students reporting higher gender-based rejection sensitivity (*M* = 4.11, *SD* = 2.85) than men students (*M* = 3.67, *SD* = 2.98; *M*_across both gender groups_ = 4.04, *SD* = 2.87). Furthermore a significant interaction effect for gender of student x gender of acting person was found, *F*(1, 371) = 17.71, *p* < 0.001, *η*_p_^2^ = 0.046, confirming Hypothesis 5. As Figure 1 shows, when the acting person was a man, women students reported significantly higher gender-based rejection sensitivity (*M* = 4.84, *SD* = 3.65) than men students (*M* = 3.6, *SD* = 3.28), *t*(95.88) = −2.69, *p* = 0.008, *d* = −0.35, while when the acting person was a woman, women (*M* = 3.38, *SD* = 2.57) and men students (*M* = 3.74, *SD* = 3.32) did not differ significantly in their gender-based rejection sensitivity, *t*(77.86) = 0.81, *p* = 0.423, *d* = 0.13. To test research Hypotheses 1 and 2 referring to the proportions of women and, respectively, men students selecting the different response options regarding gender-based relative expectations of success, we conducted a chi-squared test for women and men students separately. The tests were significant for women students: *χ*^2^(2) = 109.75, *p* < 0.001, as well as for men students: *χ*^2^(2) = 26.95, *p* < 0.001, indicating that both men and women students did not choose the three response categories with an equal probability. The largest proportion of the women students reported that women and men have the same chances of success (60.3%, *n* = 187). Confirming Hypothesis 1, while more than a quarter of the women students expected men to be more successful than women (26.5%, *n* = 82), only 13.2% (*n* = 41) believed that women would be more successful than men. Regarding our non-directional Hypothesis 2, results showed that the proportion of men students who expected better chances of success for women than for men was larger (23.8%, *n* = 15) than the proportion of men who thought that men will be more successful than women (12.7%, *n* = 8); with the remaining 63.5% (*n* = 40) expecting equal chances of success for both genders.

To test our research Hypothesis 3 according to which the proportion of women students who expect women to be less successful than men is larger than the proportion of men students, we conducted a 2 (gender) × 3 (response category) chi-square test. The chi-square test showed that men and women students chose the different response categories with different frequencies, *χ*^2^(2) = 8.10, *p* = 0.017. To examine in which of the three response categories a significant difference existed, z-tests with Bonferroni correction were conducted. These indicated a significant difference (*p* < 0.05) for the two outer response categories, but not for the middle category (“Women have the same chances of success as men in the job I aspire to”). More specifically, confirming Hypothesis 3, proportionately more women (26.5%, *n* = 82) than men students (12.7%, *n* = 8) indicated that women have worse chances of success than men in the job they aspire to. As women and men were equally likely to choose the middle category, by implication, proportionately more men (23.8%, *n* = 15) than women students (13.2%, *n* = 41) reported that women have better chances of success than men in the job they aspire to.

We performed two ordinal logistic regressions (OLR) for women and men students separately to predict relative expectations of success through hostile sexism (Hypothesis 8) and gender-based rejection sensitivity (Hypothesis 7). The use of OLR was indicated as our dependent variable was not continuous but categorically ordered (“Women have worse chances of success than men/ the same chances of success as men/ better chances of success than men”). Pearson chi-squared test, women students: *χ*^2^(506) = 526.02, *p* = 0.26; men students: *χ*^2^(116) = 112.77, *p* = 0.57, and the deviance test, women students: *χ*^2^(506) = 476.33, *p* = 0.83; men students: *χ*^2^(116) = 98.53, *p* = 0.88, indicated that the data fitted our specified models well. Further, a likelihood ratio chi-squared test showed that our models fitted the data better than the respective null models, women students: *χ*^2^(2) = 23.23, *p* = <0.001; men students: *χ*^2^(2) = 11.11, *p* = 0.004. Lastly, OLR assumes proportional odds which should be tested before interpreting estimates. In both samples the assumption of proportional odds was met as indicated by a test of parallel lines, women students: *χ*^2^(2) = 4.09, *p* = 0.13; men students: *χ*^2^(2) = 3.54, *p* = 0.17.

Results are presented in Table 1 for women students and in Table 2 for men students. In the model for women students, consistent with Hypothesis 7, gender-based rejection sensitivity predicted that women were more pessimistic regarding their own gender group’s relative success (*γ* = −0.19, *p* = <0.001, odds ratio [OR] = exp. −0.19 = 0.83). For every one-unit decrease in gender-based rejection sensitivity the odds to rate women’s success as more likely (compared to men’s success being considered equally likely or more likely) were reduced by 17% (1–0.83). Consistent with Hypothesis 8, hostile sexism did not predict women’s gender-based relative expectations of success (*γ* = 0.24, *p* = 0.17, odds ratio [OR] = exp. 0.17 = 1.27).

**Table 1:** Summary of OLR model on women’s gender-related relative expectations of success.

Parameter		*B*	*SE*	Exp(*B*)	*p*
Threshold	Expectations of success = 1	−1.39	0.39	0.25	<0.001
	Expectations of success = 2	1.67	0.40	5.29	<0.001
Gender-based rejection sensitivity		−0.19	0.04	0.83	<0.001
Hostile sexism		0.24	0.17	1.27	0.17

**Table 2:** Summary of OLR model on men’s gender-related relative expectations of success.

Parameter		*B*	*SE*	Exp(*B*)	*p*
Threshold	Expectations of success = 1	−0.04	0.70	0.96	0.96
	Expectations of success = 2	3.49	0.86	32.86	<0.001
Gender-based rejection sensitivity		0.51	0.09	1.05	0.58
Hostile sexism		0.84	0.30	2.32	0.004

In contrast, in the model for men students, reversed effects were observed: Consistent with Hypothesis 8, hostile sexism predicted stronger expectations that women are going to be more successful than men in the aspired for future job (*γ* = 0.84, *p* = 0.004, odds ratio [OR] = exp. 0.84 = 2.32). This indicates that for every one-unit increase in hostile sexism the odds to rate women’s success as more likely (compared to men’s success being considered equally likely or more likely) increased by 2.32 times. Consistent with research Hypothesis 7, gender-based rejection sensitivity did not predict men’s gender-based relative expectations of success (*γ* = 0.05, *p* = 0.58, odds ratio [OR] = exp. 0.05 = 1.05).

To test our assumption that gender moderates the relationship between rejection sensitivity (Hypothesis 7) or hostile sexism (Hypothesis 8) on the one hand and relative success expectations on the other, we calculated the interaction effect of participant genderx gender-based rejection sensitivity and the interaction effect of gender x hostile sexism on gender-based expectations of career success. Confirming Hypothesis 7, the interaction between gender and gender-based rejection sensitivity significantly predicted gender-based expectations of career success (*γ* = −0.30, *p* = 0.002, odds ratio [OR] = exp. −0.30 = 0.74). Regarding Hypothesis 8, the interaction between gender and hostile sexism predicted gender-based expectations of career success, however only marginally significantly so (*γ* = 0.52, *p* = 0.08, odds ratio [OR] = exp. 0.52 = 1.69).

## Discussion

In this research, we investigated expectations of success women and men students in the social sciences hold for their own and the other gender group’s future vocational success, as a motivational predictor of task engagement and readiness to take on difficult challenges. It is good news that the majority of the students participating in our study assumed that gender is not a predictor of success: around two-thirds of the women and men agreed that the genders do not differ *per se* in their future success. However, we also found evidence for pessimistic expectations in women. As expected, the proportion of women students who believed that men are more likely to succeed in their aspired to future job than women was greater than the proportion of women students who thought that women are more likely to succeed than men. Also as expected, proportionately more women than men students believed that women have worse chances of success than men in the job they aim for.

However, not only in women but also in men the proportion of students who thought success was relatively less likely for their own gender group was larger than the proportion of students who thought success was relatively more likely for the respective other gender group. This result is in line with previous research suggesting that both men and women are particularly sensitive to discriminatory treatment of members of their own gender group (*cf.*, Elkins et al., 2002). Women students’ pessimistic expectations possibly reflect that for them it is very salient that the proportion of women decreases with increasing career level. This interpretation is consistent with the findings by van Veelen and Derks (2022) who observed that professionals in the life, social, and behavioral sciences perceived the glass ceiling for women to be even thicker than professionals in the natural sciences, technology, and economics did. Men students’ pessimistic expectations in our study may reflect that their being in a minority position is more salient for them than the leaky pipeline that puts women at a disadvantage.

Our findings substantiate the hypothesis that the pessimistic expectation for their own gender group was predicted by gender-based rejection sensitivity in women students only. At the same time, the expectation that women will be more successful in their future job than men was predicted by hostile sexism in men students only, suggesting that it served as a self-handicapping strategy or had a self-esteem-protective function for men. However, this effect should be interpreted with caution since the interaction term of hostile sexism and gender was only marginally significant, probably because substantially fewer men participated in our study. Consistent with previous research, men exhibited more hostile attitudes toward women than women (Glick et al., 2000) and women had greater concerns about being rejected because of their gender than men (London et al., 2012). These findings are significant in that they demonstrate that even those men who choose a field of study in which women are in the majority among students, namely the social sciences, are more hostilely sexist than their women peers. Further, these results suggest that women’s higher sensitivity to gender-based discrimination does not seem to be cured by the numerical dominance of women among students, and may even be strengthened by the leaky pipeline being particularly noteworthy in disciplines with a high proportion of women on average. Going beyond previous studies on gender-based rejection sensitivity, we examined the extent to which concerns about gender disadvantage depend on the gender of the interaction partner. London et al. (2012) wanted to investigate gender-based rejection sensitivity “in competitive, historically male institutions” (p. 961). This may explain why they did not make any explicit assumptions about whether their research participants imagine a man or am women when responding to items that are ambiguous regarding the interacting partner’s gender, seemingly implying that respondents necessarily think of a man. In London et al.’s (2012) questionnaire, in only six of the 11 items the male gender of the acting person is explicitly stated (“a senior male professor”) or can be inferred (“you approach your professor to ask him…”). In the remaining five items, the gender of the acting person is not specified (“your professor,” “your boss”). No item explicitly refers to a woman. In our study we investigated the social sciences where women are well represented on average. Here, it makes sense to assume that people do not necessarily think of a man when describing a social encounter in the context of their university studies. We have assumed that in such an environment, women students are particularly anxious to be treated differently based on their gender when interacting with a man, while men students’ rejection sensitivity was predicted to be the same irrespective of the interaction partner’s gender. While a comparison of the rejection sensitivity depending on whether the acting person is a man or a woman is impossible with London’s original questionnaire, our data does allow for it. As expected, in our study women showed higher gender-based rejection sensitivity when interacting with a man than with a woman, while interaction partner’s gender did not matter for gender-based rejection sensitivity in men. A possible mechanism underlying this finding is the prototypicality of a situation where a woman gets treated in a discriminatory manner by a man because of her gender. As shown by Carlsson and Sinclair (2018), individuals are the more likely to experience an ambiguous situation as discriminatory, the more prototypical it is of discrimination, with the prototypical situation regarding gender discrimination being one in which a woman experiences a disadvantage by a man. This may explain our finding that even in a context in which women are well represented on average women were more concerned about possible gender-based rejection than men and were particularly strongly concerned when interacting with a man.

The extent to which our participants were concerned about being discriminated against because of their gender was relatively weak: on a scale from 1 to 36, women had a mean score of 4.11 and men of 3.67. How do these scores compare to the ones found in other studies? Unfortunately, rejection sensitivity has relatively rarely been described for men versus women: In most studies, it was either examined in non-marginalized populations (e.g., appearance-based rejection sensitivity in adolescents) or in relation to ethnicity/race or gender minority membership (Garthe et al., 2020, for a review). We are only aware of the studies by London et al. (2012) and Ahlqvist et al. (2013) who did compare rejection sensitivity in men and women. Here, the items applied to the world of business (e.g., you start a new job in a corporate office; you are at an important business meeting), to the university context in general (e.g., you were accepted to a graduate program), or to math and science university courses, while respondents in our study were supposed to relate all items to their own university studies – i.e., to the social sciences. Interestingly, rejection sensitivity scores were weaker in our sample than in the ones participating in the studies by London et al. (2012) who reported scores between 6.76 and 8.79 for their women participants and scores between 3.17 and 5.52 for their men participants^1^ and weaker than the score of 7.18 reported by Ahlqvist et al. (2013) for their women STEM major participants. There are several explanations why our participants were less fearful to be rejected or treated unfairly because of their gender. First, our participants had been asked to relate all items to the social sciences, while the original scale by London et al. (2012) includes scenarios from predominantly masculine environments where women face particularly strongly negative stereotypes about their group’s capabilities: business and STEM (e.g., Diekman et al., 2019; Makarem and Wang, 2020; Caleo and Halim, 2021; Shen and Joseph, 2021). A second explanation is that our participants were enrolled in the social sciences, while Ahlqvist et al. (2013) investigated STEM-students and London et al. (2012) (among others) law students: As van Veelen et al. (2019) found, women in traditionally male disciplines are not only threatened by negative stereotypes but also by being outnumbered by their male peers. Future studies should use the identical scale to measure rejection sensitivity with students from different disciplines to assess whether the social sciences are indeed a less threatening context regarding possible gender-based rejection than other academic subjects. A third explanation is that none of the scenarios provided in the questionnaire by London et al. (2012) refers to a woman as the acting person. As our findings show, the extent to which women are anxious to be rejected based on their gender depends on the gender constellation of the interaction partners in the respective situation, with women students being more concerned about possible gender disadvantage when imagining an interaction with men staff or peers.

### Limitations

There are several limitations of our study that must be considered in the interpretation of our findings. We surveyed our participants’ subjective expectations regarding success in their future job without considering the extent to which men and women might actually have different chances in different professional fields in the social sciences. Further studies should examine the relationship between students’ subjective expectations of success and actual relative career opportunities for women and men in different occupational domains. In addition, we asked about gendered success expectations in future job, so possibly some students may have been thinking about careers outside of social science. Furthermore, we investigated only two potential predictors of gender-based relative expectations of success in our study. Thus, it is quite likely that the expected success depends on other relevant predictors too, such as the subjective assessment of the gender group’s competence in the field or different career aspirations and life plans that are attributed to men and women. It is also possible that we missed including other relevant variables on the individual level in the survey (e.g., self-efficacy beliefs). Further research in this area should consider these possibilities. Likewise, further research should investigate career related expectations of success and their predictors in people who do not feel they belong to any of the two binary gender groups or who identify as non-binary. Our hypotheses related exclusively to students identifying as either women or men and to expectations of success regarding women’s and men’s future success. There were several reasons for this. We had expected that the group of students identifying as non-binary would be so small that their data could not have been analyzed by parametric statistical techniques, and this proved to be so in our sample. Also, we would have been investigating a different research question had we asked students how they rated the success of their own gender group relative to the success of the group identifying as non-binary. By asking this question, we would have examined possible prejudices of men and women toward this gender group, which presumably depend on different predictors than those we examined (gender-based rejection sensitivity and sexism toward women). Due to the small number of people identifying as non-binary in our sample, we were unfortunately unable to examine their data.

### Implications of our findings

What are the implications of our research findings for how to improve the motivational situation of women and men students in the social sciences? Women students’ concern of being rejected due to gender suggests that the environment of their university studies is not identity safe: With increasing gender-based rejection sensitivity we found women students to be less optimistic regarding their own gender group’s relative professional future success. Van Laar et al. (2019) describe identity safety as a context in which people do not feel threatened regarding any aspect of their personal identity and thus do not need to regulate threats. Factors that promote identity safety include the conveyance of the feeling that one’s social group is accepted and valued, as communicated through the diversity climate of the organization – be it the workplace or an educational institution, such as a university: An organization with a positive diversity climate signals to be open toward and to welcome various social groups (Van Laar et al., 2019). A subtle factor by which students can gauge how welcome women actually are in their field of university studies is the representation of women among high-status and influential members of the university, specifically professors and assistant professors or highly placed representatives of the administration. An increase in women’s representation in such high-ranking positions at the university should diminish potential triggers of identity threat for women students and hence have a favorable effect on their expectations regarding their own opportunities to attain a professional position with high social status within their field of study.

According to Van Laar et al. (2019), another indicator of identity safety is instrumental or emotional support provided by the organization, for instance by representatives of the university in positions of authority and power. Our results show that women students are less anxious to be rejected because of their gender in social interactions with a woman than with a man. A stronger representation of women among high-status academic staff would make it more likely for women students to encounter women in high-stakes academic settings (e.g., an admission interview or an oral exam) and should therefore reduce gender-based rejection sensitivity. What is more, high-status women in the university setting serve as ingroup role models for women students. Ingroup expert models have been shown to mitigate the effects of negative stereotypes on stigmatized individuals as they invalidate the assumption that individuals affected by the stereotype cannot succeed: They have a successful career to their credit, despite the obstacles that members of the respective group need to overcome (Marx and Roman, 2002; Steele et al., 2002; Liu et al., 2021). Against this background, Dasgupta (2011) proposes that ingroup expert models inoculate stigmatized individuals not to experience threat and self-doubt in high-stakes environments.

Our finding that men students considered their own gender group’s relative future success less likely the more hostile their attitudes toward women were suggests gender-based zero-sum thinking, i.e., the belief that women succeed at the expense of men (Kuchynka et al., 2018). As Van Laar et al. (2019) emphasize, this finding suggests that identity safety needs to be assured not only for members of negatively stereotyped groups, but also for members of the dominant or majority group, not to make them feel that the organization’s diversity efforts put their own group at a disadvantage. In the social sciences, where men students are outnumbered by their women student peers, an all-inclusive environment (Emerson and Murphy, 2014) providing identity safety for all students signals that men are just as valued and welcome as women. Only to the extent that zero-sum beliefs about the professional success of men and women can be reduced among students of both genders can women and men students also be expected to affirm measures for more gender equity, such as an increase in the representation of women in high-status positions at the university.

## Data availability statement

The data supporting the conclusions of this article will be made available by the authors on request.

## Ethics statement

The studies involving human participants were reviewed and approved by Ethikkommission des Fachbereiches Erziehungswissenschaft und Psychologie der Freien Universität Berlin. Written informed consent from the participants’ legal guardian/next of kin was not required to participate in this study in accordance with the national legislation and the institutional requirements.

## Author contributions

KO, MR, and BH contributed to the design and implementation of the research, to the analysis of the results and to the writing of the manuscript. All authors contributed to the article and approved the submitted version.

## Funding

We acknowledge support by the Open Access Publication Initiative of Freie Universität Berlin.

## Conflict of interest

The authors declare that the research was conducted in the absence of any commercial or financial relationships that could be construed as a potential conflict of interest.

## Publisher’s note

All claims expressed in this article are solely those of the authors and do not necessarily represent those of their affiliated organizations, or those of the publisher, the editors and the reviewers. Any product that may be evaluated in this article, or claim that may be made by its manufacturer, is not guaranteed or endorsed by the publisher.

## Footnotes

^1^While participants in London et al.’s (2012) Study 5 were law students no information is provided about the fields of study of the students participating in the other studies.
